# The characteristics of general practice and the attractiveness of working as a GP: medical students´ views

**DOI:** 10.5116/ijme.530e.3e4e

**Published:** 2014-03-15

**Authors:** Bjorn Landstrom, Bengt Mattsson, Per Nordin, Carl E. Rudebeck

**Affiliations:** 1 Department of Public Health and Community Medicine, Sahlgrenska Academy, University of Gothenburg, Sweden; 2 The Skaraborg Institute, Stationsgatan 12, SE-541 30 Skovde, Sweden; 3 Esplanaden Health Care Centre, Sweden and Institute of Community Medicine, Tromso University, Norway

**Keywords:** General practice, medical students’ views, recruitment, medical education

## Abstract

**Objectives:**

The aim of the study was to investigate medical students´ views on general practice based on their experiences in training, and to find out whether there were certain views associated with the intention to become a GP.

**Methods:**

A questionnaire, based on our earlier studies about GP working behaviour, was handed out to medical students in terms 1, 3, 5, 7, 10 and 11 of undergraduate studies in Gothenburg, Sweden. The analysis comprised statistical descriptions and comparisons.

**Results:**

The students regarded general practice positively. They found the work environment good, the GP´s awareness of patients’ living conditions necessary, and that GP work requires medical breadth. The status of the GP in the medical profession was not considered high. One-fourth of the students strongly agreed with the possibility of a future as a GP. This attitude was statistically associated with support to the statements that general practice offers a good work environment and should be a major component in undergraduate training. Students with a negative attitude to working as GPs were also negative to having a major component of general practice in undergraduate training.

**Conclusions:**

Medical students with a positive stated attitude towards becoming GPs support changes in undergraduate training to include more general practice. The risk of increasing a negative attitude should be considered when changes are discussed.

## Introduction

Internationally the domination of biomedical theory and hospital experience in clinical training is gradually being balanced with an increasing input of general practice and early experience of patient consultation. During the past ten years training programmes focusing on primary care and located at GPs’ practices have been initiated in both Europe and the USA.^1^^,^^2^ There is an emerging awareness of the importance of the context of learning.^3^ General practice has gradually become more articulate internationally as to its role in health care, mirrored through the regular documents from the GPs’ world organization.^4^ The recruitment of new GPs, however, has become more problematic.^5^^,^^6^^,^^7^

Internationally, the results of studies of students’ views concerning general practice are somewhat conflicting. In a study from Ohio, students’ attitudes approached those of GPs towards the end of their education. This increasing agreement however coincided with a decrease in the students’ interest to become GPs.^8^ The opposite was found in an English study. Here, attitudes were more positive in the final phase of their education, especially for female students. A plausible explanation was a well-adapted apprenticeship included in the general practice training.^9^ Likewise, in a study from Aberdeen, medical students’ attitudes to general practice were more positive towards the end of the training.^10^

Looking at single elements that have an impact on medical students’ attitudes towards general practice, there is a clear but short-term effect of positive experience of supervision, while negative experience appears to make students more critical.^11^ The personal enthusiasm shown by supervisors/GPs is, not surprisingly, quite important.^12^ Looking at factors that actually increase the attractiveness of general practice, it has been concluded that integrated changes throughout the education and training period, with general practice all along the pipeline, seemed to have the greatest impact.^13^ With such a design, the students get the opportunity to attain a deeper and concrete understanding of the concepts and ideas describing general practice theoretically. Before their eyes they see the patient-centred version of medicine; the integration of the personal and context bound to the abstractions of biomedicine in dialogue and attentive listening.^14^ Exploring medical students’ views on general practice should not just be about preferences and general attitudes but also about their perceptions of what actually takes place between patient and doctor. They may very well appreciate what their GP tutors have to show them, but still plan to become paediatricians or surgeons themselves. In apprenticeships students also get a fair idea about the working terms of GPs, which might be crucial as to their considering general practice as a specialty.

Medical education in Sweden is eleven terms. Changes to give more general practice in the education in Gothenburg were introduced ten years ago and the curriculum was much the same in 2011. The teaching in general practice is divided into three courses. An early professional contact course (TYK=tidig yrkeskontakt) includes eight days in a health care centre- plus a number of seminars- during the first two years. The “consultation course” where students train in taking medical history and clinical examination, under the supervision of GP tutors, is given in the fifth term. The final year students attend a two-week course of unselected patients with different problems at the health care centres. The training is followed up with ensuing reflections. Thus students have GPs as tutors on average for one week per term all through the curriculum.^15^

In the ongoing international discussion about medical education, its context of learning and the increasing responsibilities put on general practice in fostering basic clinical competence in the students, the discussion in Sweden is not different from many other countries. Thus experiences from the Swedish medical undergraduate training have relevance for medical education in other countries as well. Therefore, given the input of general practice to medical training at the University of Gothenburg, and with the recruitment of future GPs in mind, the aim of the study was to investigate medical students´ views of general practice and to find out whether certain views were associated with the idea of general practice as a possible career choice.

## Methods

### Study design

The study is explorative and uses a questionnaire. The statements in the questionnaire were constructed from our earlier studies and were focused on key features of general practice as they were identified by GPs and students.^16^^,^^17^^, 18^ Our first study showed that professional development can be described as a transition of deliberate, favourable strategies into the GP´s personal style. The statements about work environment, how varied GP work is, the importance of dialogue and the importance of the patients´ living conditions derive from this study.^16^

The second study explored empirically how theory and practice are integrated within the work of GPs. The statements derived from this study are about breadth of medical knowledge, that GP patients are not selected by disease and how seriously ill the patients are. The study also contributed to the statement about living conditions.^17^ The third study which was about final-year medical students´ perspectives of GPs´ competencies showed that the understanding of, and confidence in general practice is considerable. This study contributed to the statements about importance of dialogue, medical breadth, that GP patients are not selected by disease and the importance of living conditions.^18^ The statements about status, general practice in undergraduate training and a possible GP career were added according to the aim of the study.

The statements were discussed among the four authors until we agreed that the questions were feasible, understandable and unambiguous. The statements were then modified after tests on younger doctors and medical students. The questionnaire finally consisted of ten statements about students´ views, with four alternatives. Age and sex were also asked for. It was considered to be easy to answer.

### Participants

Undergraduate medical students in Gothenburg at all levels were selected in this study. We made a strategic selection, every second term and the beginning of term 10, to represent all stages of the curriculum. In winter 2011 there were altogether 576 medical students in terms 1, 3, 5, 7, 10 and 11 in Gothenburg, which were all included in the study.

### Data collection

Our study was welcomed by heads of medical training in Gothenburg, which gave us an entry into each course. One of the researchers (BL) visited scheduled lessons on each of the six studied terms and in agreement with the lecturer informed the students about the study. The students were informed that participation was voluntary and confidential (we had no access to the course register). The questionnaire, ten statements with four alternatives, was then handed out to the students and answered anonymously and voluntarily. In Sweden there is no requirement for ethical committee approval for this kind of study.

### Data analysis

The study was analyzed in two parts. The first part was descriptive with frequencies, means, medians, confidence intervals and contingency tables. The second part consisted of pair-wise comparisons using χ^2^ test as well as multiple comparisons using logistic regression. In the regressions, categorical explanatory variables were analyzed using dummy-variable technique. The significance level and the confidence level were chosen to be 5% and 95% respectively.^19^ Statistical comparative tests were made for investigating whether certain attitudes were connected with the intention to become a GP.

### Results

Out of 576 students 513 attended and 508 responded. Of these 287(56.5%) were women and 214(42.1%) were men, with 7(1.4%) of the students not stating sex. The response rate was thus 88% (95% term 1, 94% term 3, 74% term 5, 94% term 7, 85% term 10 and 87% term 11). The lower response rate, 74%, in term 5, was due to the fact that the lesson chosen was not mandatory and fewer students attended. The mean age for women was 25.1 years [min age 18, max age 50] SD=4.7 and for men 26.3 years [min age 19, max age 46] and SD=5.6.

The work environment in general practice was assessed by the students as good, 86% agreeing completely or to a large extent (see Table 1). They were aware of the importance of knowledge about patients’ living conditions and that medical breadth is required (96 and 99% respectively). The GP was not thought to have high status in the profession (only 16% agreed completely or to a large extent with the survey question about high status). A quarter of the students agreed completely with the survey question about the possibility of working as a GP in the future. No sex differences were found.

**Table 1:** Distribution of survey statements among medical students in terms 1, 3, 5, 7, 10, 11(N=508)

Survey questions	Alternatives %				No. responses
	agree completely	agree to a large extent	agree to a lesser extent	do not agree	
GP work offers good work environment	22.7	63.2	13.3	0.8	503
GP work will become monotonous over time	3.8	24.9	53.0	18.3	502
For the GP dialogue is the most crucial aspect of diagnosis and treatment	31.4	59.3	8.9	0.4	506
GP work requires breadth of medical knowledge	81.5	17.5	0.8	0.2	508
For a GP being aware of the patients’ living conditions is necessary	50.6	45.1	4.1	0.2	508
For a GP patients are not selected by disease	19.7	51.1	27.6	1.6	493
Within the GP´s work, one meets mostly patients who are not seriously ill	7.3	43.5	40.9	8.3	506
The GP has high status within the medical profession	1.2	15.9	59.2	23.7	503
General practice should have a major place in undergraduate training	30.7	56.7	12.6	0	501
I regard working as a GP as a possible choice for the future	25.0	39.3	26.2	9.5	504

Table 2 reports the distribution across the terms of the students who answered “agree completely” (alternative 1) to the statements. The statement “I regard working as a GP as a possible choice for the future” with answer alternative “agree completely” is the positive alternative in our construction of the binary outcome variable used for multiple comparisons.

**Table 2:** Relative frequencies of the answer alternative “agree completely” to survey statements across the terms (N=508)

Statement	Agree completely % per term						all terms
	1	3	5	7	10	11	
General practice should have a major place in undergraduate training	22.9	29.2	29.2	21.1	48.8	37.3	30.7
I regard working as a GP as a possible choice for the future	18.4	25.8	24.7	21.1	34.6	27.7	25.0
Number of students responding	98	97	73	92	81	67	508

In term 1, a significantly lower proportion of the students agreed completely with “dialogue as the most crucial part of diagnosis and treatment”, 18% (95%CI [11-27%]), compared to students in terms 7, 10 and 11, 39% (95%CI [33-45%]). The confidence intervals do not overlap (see Table 2).

When comparing the answers to the statement “I regard working as a GP as a possible choice for the future” we found statistical associations (χ^2^ analysis) with the following factors (statements 1,2,4,5,9): good work environment (p=0.0001), variation in work (p<0.0001), breadth (p=0.001), knowledge of the patient’s living situation(p=0.044) and major part of GP training (p<0.0001). There was also an association with rising student age (p=0.004). No such associations were found with unselected patients, severity of disease, importance of patient-doctor dialogue, status (statements 2, 6, 7, 8) nor with sex and term. The outcome variable used in the logistic regression was designed to compare the students that were most positive (“agree completely”) to the statement, “I regard working as a GP as a possible choice for the future” with the rest of the students. In the logistic regression, dummy variable technique was used to cope with non-equidistant categorical explanatory variables. Some of the combinations of the explanatory variables got zero frequencies and therefore odds ratios could not be calculated.Two factors remained as independently associated with the outcome variable: good work environment (in its last alternative with no significance, “do not agree”, there were only two answers) and major share of GP training (statements 1 and 9). Statement 5, awareness of patients´ living conditions, was nearly significant at a 5%-level. Age and sex were not associated. An overall joint test of the parameter estimates of “good work environment” (χ^2^ =19.0, p<0.001) and “major share of GP training” (χ^2^ =59.7, p<0.001) was significant in the proposed model.The students that “agree completely” on working as a GP stated “agree completely” in 62% (95%CI [53-71%]) to the statement that general practice should have a major share in undergraduate training compared to 31% (95%CI [27-35%]) among all students. Among the students that “do not agree” on working as a GP 4% (95%CI [0.5-15%]) answered “agree completely” to the same statement that general practice should have a major place in undergraduate training.

## Discussion

The medical students in this study responded that they regarded general practice positively. Two thirds stated that they were considering a career as a GP (agree completely and to a large extent). This is in line with the results from a survey study in Gothenburg (terms 8, 9, and 10).^20^One-quarter of the students agreed completely about considering a career as a GP. Factors statistically associated (logistic regression) with this were good work environment and wanting a larger share of general practice in training. These results were in line with the results of the Shadbolt study^13^ and indicate the importance of training in general practice at returning levels during the education. Students might see advantages of more general practice in their education irrespective of their planned career, due to the broad scope of the specialty “general practice”. However, the students least interested in working as GPs were also negative to a larger share of general practice in medical education.The students regarded the doctor-patient dialogue as crucial for diagnosis and treatment. This may reflect an influence of the general practice perspective on nearly all students irrespective of career plans; or the fact that this view is also entertained in other clinical courses. Irrespective of cause, this finding is encouraging as to the future quality of doctor-patient communication in the clinic as a whole.Doctors are role models for medical students. GP teachers with negative attitudes influenced students negatively about general practice.^21^ GPs viewed as good mentors strongly influenced the willingness to choose that specialty.^22^ Therefore it may seem problematic for general practice that in our study the status of the GP was thought to be low. However, our previous study shows GPs mostly as positive role models when actually observed by students, and is supported by the finding that the personal enthusiasm of GP supervisors has a positive effect on the students´ attitudes to the specialty.^18,^^12^If motives for medical students interested in general practice are divided into two groups, one is mainly ideologically-oriented towards general practice, while the other stresses working conditions. Individual aspects such as “personal ambition”, “future perspective” and “work-life balance” were important, as was “patient orientation” when investigating factors favouring a career as a GP among medical students.^23^ Our own and others’ studies^16^^, ^^21^^, ^^24^ do not contradict such a view.Curriculum development by inserting general practice throughout the medical education is the most effective way of making general practice more attractive.^13^ In our study the association between interest in becoming a GP, and wanting a major share of general practice in undergraduate training, supports this. Changes in this direction would be important for recruiting more GPs in the future. We have identified a group of students who are negative both to becoming a GP and to inserting more general practice into the training. Radical changes in the curriculum might therefore cause this negative group to increase.

**Table 3:** Results of the logistic regression*

Explanatory variables		Odds Ratio	Std. Error	95% Conf. Interval	
				lower	upper
Good work environment	Agree to a large extent	0.38	0.10	0.22	0.64
	Agree to a lesser extent	0.23	0.10	0.10	0.55
	Do not agree	3.71	5.61	0.19	71.7
Undergraduate training	Agree to a large extent	0.18	0.05	0.11	0.30
	Agree to a lesser extent	0.10	0.05	0.04	0.29

*Results are adjusted for age and sex. Outcome variable is defined as the most positive students against the rest, to the statement “I regard working as a GP as a possible choice for the future”.

Attractiveness of general practice, in this study expressed by the low proportion of students who hold GPs as having high status, may not be explained mainly by the medical education. The organization and working conditions in general practice are probably more important explanations. The main implication of this study for medical education is that more training in general practice is required if more young doctors are to make general practice their choice. Another implication is that working conditions in general practice are more important than the status of GPs within the medical profession.The questionnaire statements are based on the results of the qualitative studies with both GPs and students, thereby creating better provisions for obtaining validity and reliability to the questionnaire.^16^^,^^17^^,18^ Aspects of general practice mentioned should be recognized by the respondents not just semantically. The pilot study indicated that the questionnaire was understandable and consistent. The statistic results indicate that the statements of the questionnaire were discriminating and that the attitudinal groups among the students identified have to be accounted for in the curriculum planning. We set out with four answer alternatives for each statement but found that in several statements the students tended to choose alternative 2 or 3. By linking the agreement “agree completely to having a major share” to the statement “general practice should have a major share…” with the agreement to the statement “I regard working as a GP as a possible choice for the future” (statement 10) we hoped to select a group of students that were more determined to become GPs. It should be remembered, though, that the questionnaire mirrors only the students´ views at the moment and provides no accurate prognosis for their future choice.Comparisons between different terms is motivated since no major changes have been undertaken in the undergraduate training in Gothenburg during the past ten years; neither has the admission system undergone major changes. In that sense, our study population represents all medical students in Gothenburg at the time of the study. In other medical centres in Sweden the situation is rather similar concerning undergraduate training in medical education. There are no indications that the backgrounds of the medical students differ much among the universities in Sweden.

## Conclusion

Positive attitudes to general practice among medical students are based on the recognition of its key features in the real setting. Medical students with a positive stated attitude to becoming GPs support changes in undergraduate training to include more general practice. The risk of increasing the negative group should be considered when changes are considered. There may exist a critical level above which the reforms to promote general practice in medical training may become counterproductive.

### Limitations

This paper evaluated data from one medical college and only from one half of the medical student population. Analysis from other medical colleges may produce different results. Factors that limit generalization for other countries are the differences among the systems of primary care, and their roles in the whole of health care. The fact that GPs are involved on a broader scale in medical education internationally suggests, however, that our findings may apply in other settings.

## 

### Conflict of Interest

The authors declare that they have no conflict of interest.
